# Mechanical Properties of a Sustainable Low-Carbon Geopolymer Concrete Using a Pumice-Derived Sodium Silicate Solution

**DOI:** 10.3390/ma17081792

**Published:** 2024-04-13

**Authors:** Jonathan Oti, Blessing O. Adeleke, Francis X. Anowie, John M. Kinuthia, Emma Ekwulo

**Affiliations:** 1 Faculty of Computing, Engineering and Science, University of South Wales, Pontypridd CF37 1DL, UK; jonathan.oti@southwales.ac.uk (J.O.); fransanowie@yahoo.com (F.X.A.); john.kinuthia@southwales.ac.uk (J.M.K.); 2 Department of Civil Engineering, Faculty of Engineering, Rivers State University, Porth Harcourt PMB 5080, Nigeria; eoekwulo@gmail.com

**Keywords:** geopolymer, alkali alkaline activator, silica fume, consistency, compressive strength, pumice, aluminosilicate, GGBS

## Abstract

A geopolymer is an inorganic amorphous cementitious material, emerging as an alternative sustainable binder for greener concrete production over Ordinary Portland Cement (OPC). Geopolymer concrete production promotes waste reuse since the applicable precursor materials include agricultural and industrial waste that requires disposal, helping to reduce waste in landfills and ensuring sustainable environmental protection. This study investigates the development of an environmentally friendly sodium silicate alternative (SSA) derived from pumice powder (PP) in place of a commercial Na_2_SiO_3_ solution at a 10 M concentration. Six concrete batches were produced at alkaline/precursor (A/P) ratios of 0.1, 0.2, 0.3, 0.4, and 0.5. The geopolymer mix AF4, with an A/P ratio of 0.4, became the optimum geopolymer concrete design; however, it recorded lower compressive, tensile splitting, and flexural strengths, respectively, against the control OPC concrete. The geopolymer formulations, however, obtained 28-day-hardened concrete densities comparable to the control concrete. The 28-day compressive strength of the OPC concrete was 29.4 MPa, higher than the 18.8 MPa recorded for AF4. However, the 56-day strength of AF4 improved to 22.4 MPa, an around 19% increase compared to the 30.8 MPa achieved by the control mix on day 56, having experienced only a 5% strength increase. The low mechanical performances of the geopolymer formulation could be attributed to extra water added to the original geopolymer design to improve the workability of the geopolymer mix. Therefore, the SSA alkaline solution using PP showed some potential for developing geopolymer concrete for low-strength construction applications.

## 1. Introduction

The global demand to cut down carbon in concrete production and reduce greenhouse gas (GHG) emissions and their effects on the climate has challenged concrete technologists to move towards sustainable net-zero concrete solutions [1]. The versatility of concrete, which can be moulded into any shape, and its resistance to severe loading and abrasion under service, make it a major construction material for various infrastructures over other alternatives [2]. Moreover, traditional hydraulic binder cement used for concrete production accounts for about 7% of global carbon dioxide (CO_2_) emissions, yet the intensity of direct CO_2_ emission for cement production between 2015 and 2021 increased by around 1.5% annually. In contrast, the global expectation is to have it reduce by 3% per annum up to 2030 to achieve the 2050 net-zero emission goal [3,4]. This is due to the significant challenge the cement industry faces between reducing CO_2_ emissions by cutting down production and meeting the increasingly high “cement-concrete” product demand from end-users [3,4,5].

However, cement replacement with industrial waste materials has not led to a substantial reduction in the carbon and energy involved in concrete production because some of the supplementary cementitious materials used are produced similarly to OPC, with high carbon amounts involved [6,7]. Using concrete binder produced from an alkaline-activated material (AAM), also termed as geopolymer, an inorganic binder, is proving to be a more sustainable alternative to traditional concrete incorporated into industrial waste (e.g., fly ash (FA), slag, calcined clay, and silica fume (SF), among other materials with pozzolanic properties); thereby, reducing cement content in concrete has shown a comparable and sometimes favourable mechanical performance over OPC concrete [2,3,8,9,10,11]. An AAM is either an alkaline-based activated binder with a composition of Na^+^ or K^+^ in an aluminosilicate-based system known as a geopolymer or alkaline-activated slag with a comparatively higher calcium oxide (CaO) composition than the base (precursor) materials used for the geopolymer [12,13,14]. The inorganic aluminosilicate polymer gel binder for geopolymer concrete is produced from industrial waste precursors like pulverised fuel ash (PFA), ground granulated blast-furnace slag (GGBS), silica fume (SF), metakaolin, and agricultural waste materials that contain adequate aluminium and silica and can be activated with mostly alkalis of hydroxide and silicate solutions [15]. In addition, geopolymer concrete utilizes the process of the polycondensation of silica and alumina precursors and alkali to generate structural strength, with the atomic structure of the polymeric gel displaying a similar structure to that of the zeolite material [1,16]. According to Duxson et al. [17] and Singh et al. [18], geopolymer concrete has a lower carbon footprint than conventional concrete. Shobeiri et al. [19] corroborated this claim and suggested that material type and composition in a geopolymer design influence the carbon emission rate. Likewise, Turner and Collins [20] indicated that the CO_2_ footprint for geopolymer concrete is about 9% less than that for PC concrete. Imtiaz et al. [21] used the midpoint approach to determine geopolymer concrete’s life cycle impact assessment, showing that geopolymer concrete reduces the GWP by 53.7% over PC and further reduces the acidification potential and photochemical oxidant formation. Hence, attention is shifting to using geopolymer as a new binder material for greener concrete production over OPC concrete [1,14,21,22].

Geopolymers’ adoption into the built environment provides an avenue to utilize waste streams and reduce net heavy metal emissions into the environment [23]. Indigenous and some agricultural waste like palm oil fuel ash (POFA) and rice husk ash (RHA) have also found considerable use for geopolymers, making it feasible to develop geopolymer concrete in every part of the world to safeguard landfills and reduce environmental impact everywhere. Regarding alkaline activators, they are highly corrosive, provide huge handling challenges for large-scale concrete production [24], and are also the main contributors to the embodied energy and CO_2_ emissions of geopolymer concrete [25]. Environmental impact studies show that alkali activators reduce the sustainability of geopolymer concrete [20,26]. The cost of commercially produced alkaline activators is also a disincentive for the sustainable promotion of geopolymer concrete [27]. The commercially produced sodium silicate (Na_2_SiO_3_) alkaline solution is associated with high carbon emissions and, thus, is not environmentally sustainable for geopolymer concrete production [27]. Therefore, the CO_2_ cost from the elevated-temperature calcined step in PC concrete, avoided when geopolymer concrete is adopted, is reintroduced in a way through the use of alkaline activators with a high embodied energy [23]. Therefore, substituting the commercially produced Na_2_SiO_3_ alkaline activator with a more sustainable alternatively derived from Na_2_SiO_3_ leads to a lower global warming potential (GWP) in geopolymer concrete. Ongoing attempts have been made to develop geopolymer concrete using a sodium silicate alternative (SSA) alkali solution derived from industrial and agricultural waste such as rice husk and silica fumes having a high silica content [28,29,30,31,32,33]. Billong et al. [29] compared the mechanical performance of metakaolin and GGBS-based geopolymer paste using commercially produced sodium silicates and an SSA derived from SFs mixed in a concentrated NaOH solution. The results indicated a slightly better performance in some mechanical properties, like density and compressive strength, of the geopolymer paste with the derived SSA and sodium hydroxide as the activating reagents. Tong et al. [34] also investigated the mechanical and microstructural performances of an FA-GGBS blend geopolymer binder activated with NaOH and an SSA prepared from RHA and a concentrated NaOH solution. The compressive strength and setting time results of the geopolymer mortar were comparable to the ones produced from commercial Na_2_SiO_3_. In addition, a microstructural analysis also confirmed that the derived SSA had the required amount of silicates in a reactive form.

Pumice stone is a low-density, lightweight rock formed from rapidly cooling molten lava trapping gas bubbles during volcanic action [35]. Pumice stone is used in the construction industry as a lightweight aggregate, and the powder is often used as a partial cement replacement due to its pozzolanic properties [36]. In geopolymer development, pumice stone is used in its powdery form (pumice powder—PP) as either a partial replacement of another precursor-based geopolymer or as the main precursor material due to its richness in Al and Si content [37,38]. Nematollahi et al. [39] confirmed the feasibility of a one-part FA geopolymer activated with NaOH and Na_2_SiO_3_ in the powder form and obtained a compressive strength of 29 N/mm^2^, which was comparable to the two-part formulation when NaOH and Na_2_SiO_3_ alkaline solutions were employed as the activators. However, Gokçe et al. [40] discovered conflicting mechanical strengths from using a Na_2_SiO_3_ activator for one-part and traditional two-part geopolymer formulations. One-part geopolymer concrete production, using anhydrous sodium and silicate alkalis hydroxide, enables easy handling [24]. Likewise, Provis and Van Deventer [41] affirmed that producing a geopolymer with a solid activator could generate a lower early strength compared to using the solution because of the possibility of a pre-reaction with the precursor during dry-mixing due to its hygroscopic nature and slowness in developing its alkalinity during mixing. To investigate the ambiguity of geopolymer concrete design (one and two-part formulations) due to the complexity in geopolymer mix formulation/performance and employ the environmental use of PP as part of the proposed environmental geopolymer mixes, this study resorted to the conventional two-part geopolymer concrete design to ascertain the viability of producing an SSA derived from pumice powder (PP) with a high silica content and the engineering properties of the produced geopolymer concrete as opposed to a commercially sourced SSA.

## 2. Materials

**Figure 1:**
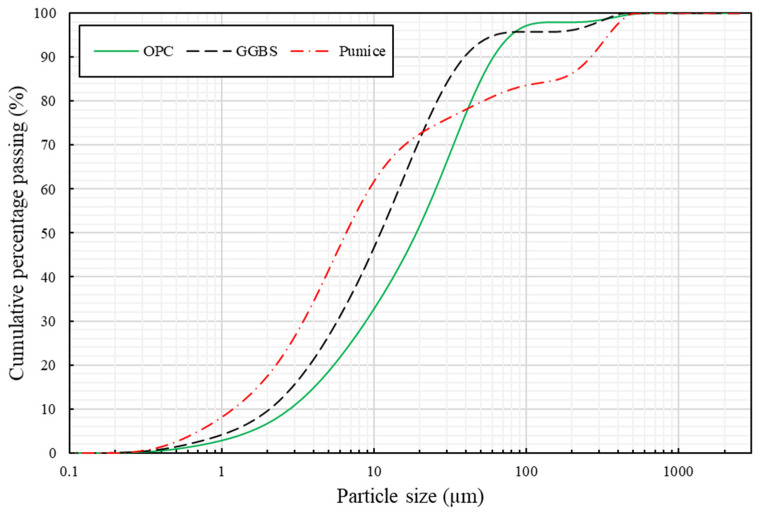
Particle size distribution for the binder materials.

Ordinary Portland Cement (OPC) was used to produce the control concrete mix. The OPC used was manufactured and supplied by the Hanson Heidelberg Cement Group, Maidenhead, UK in accordance with BS EN 197-1:2011 [42]. Ground granulated blast-furnace slag (GGBS) was used as the precursor material for developing the geopolymer concrete. GGBS is a by-waste product generated from the steel-making industry. GGBS is a latent hydraulic material that can react directly with water, requiring alkali activator for hydration [43]. The GGBS used was produced and supplied by Tarmac, UK, part of the CRH company, in accordance with BS EN 15167-1:2006 [44]. The oxide compositions of OPC and GGBS are shown in Table 1. The sodium hydroxide (NaOH) solution was prepared from NaOH pellets, manufactured and supplied by Fishers Chemical Ltd., Loughborough, UK. The NaOH white pellets had a molecular weight of 40 g/mol and a pH value of 14. NaOH was chosen over KOH with a higher alkalinity level, but studies have shown that NaOH demonstrates a greater capability to release more aluminosilicate monomers than KOH [17]. The NaOH and pumice powder were mixed to derive the sodium silicate alternative (SSA). Pumice powder (PP) is produced from pumice rock, a product of explosive volcanic eruptions. The PP used was procured from a local supplier in Cardiff, UK. The particle distribution curves for the binder materials are shown in Figure 1 and indicate a rather higher proportion of fine particles for pumice in relation to OPC and GGBS. The chemical composition (average oxides) and physical properties of PP are shown in Table 1 and Table 2.

A natural limestone aggregate of two particle sizes, 10 mm and 20 mm, was used for the concrete according to BS EN 12620:2002+A1:2008 [46]. The fine aggregate used for this study was a natural river sand with 99.2% particle, passing through a BS sieve size of 2 mm. The fine aggregate was supplied by Jewson Ltd. in accordance with the appropriate standard [46]. The geometrical, mechanical, and physical properties of the aggregates are shown in Table 3 below.

## 3. Methods

### 3.1. Mix Design

Six different mix designs were considered, including five geopolymer mix designs, and one (1) control mix design using OPC as the binder. A concrete design ratio of 1:2:3 was adopted for both the control OPC concrete and the geopolymer concrete mixes. The control concrete design mix materials were batched by weight in compliance with BS EN 206:2013+A1:2016 [47]. The designated quantities of the NaOH (SH) and Na_2_SiO_3_ (SSA) solutions (two-part mix design), the GGBS precursor material, water, and coarse and fine aggregates were batched by weight and stored in separate containers. The alkali activators were in an SH:SSA ratio of 1:1.

Water/binder ratios of 0.55 and 0.44 for the OPC control mix and the geopolymer mixes were the water requirements for producing the concretes. An extra 0.5 litres of water were added to the geopolymer concrete formulations, and, after a trial, concrete produced with the original 0.44 water/binder ratio produced an unworkable concrete [48]. This was to avoid using concrete plasticizers, resulting in the production of concrete of a higher cost and GWP. The designed total mass of the binder in the geopolymer concrete, i.e., the sum of the masses of the precursor and the alkaline activators used, was set to equal the design mass of the cement binder in the concrete control mix. The alkaline/precursor (A/P) ratio varied from 0.1 to 0.5 for the geopolymer mix designs, while the coarse and fine aggregate mass was the same for all the mix designs. Table 4 presents the material quantities used for control and geopolymer concrete production.

### 3.2. Test Alkaline Solutions’ Preparation

A good alkaline activator for geopolymer production is any soluble substance that can supply alkali metal cations, raise the reaction’s pH, and facilitate the dissolution of the aluminosilicate precursor materials [41]. Sodium and potassium hydroxides and their silicates are common alkalis used to synthesize geopolymers, but the alkalis of sodium are often the preferred choice due to their low viscosity, wide availability, and lower cost [1]. Alkaline hydroxides, alkaline silicates, and a mixture of the two are the usual alkaline activators for alkaline-activated geopolymers [14].

Firstly, the geopolymer’s alkaline activation solution was prepared from NaOH pellets and pumice. In this step, a 10 M NaOH stock solution was prepared by mixing 2800 g of NaOH pellets in 7000 g of deionised water at an ambient temperature of 20 ± 2 °C for 24 h. The molar ratio of the dissolution between the NaOH solid and water to produce the aqueous NaOH solution was 1:1. A 10 M NaOH solution was adopted because it is a common molar concentration used to synthesize geopolymer concrete to create optimal mechanical properties [29]. The NaOH solution was then stored in a safe, closed container for over an hour to ensure a complete mixture. Secondly, the SSA solution using PP was designed using Equation (1) according to Adeleke et al. [30] and Billong et al. [29] and prepared as follows: 2958 g of PP was mixed in 3500g of the initially prepared NaOH solution at an ambient temperature of 20 ± 2 °C and kept for at least 24 h before use. This ensured a thorough mixing of the PP in the NaOH solution and the dissolution of silica in an alkaline environment at ambient temperatures to produce the desired pumice-derived sodium silicate solution. The determination of the amount of PP required in the reaction with a 10 M NaOH solution to produce the SSA solution was computed using the 71% silica content from the PP.

(1) $$2SiO_{2}+2NaOH\to Na_{2}O({{SiO}_{2})}_{2}+H_{2}O$$

### 3.3. Concrete Specimens’ Preparation and Testing Methods

The control OPC concrete was produced in accordance with the appropriate British Standards. The geopolymer concrete was produced by mixing dry materials, including the precursor (GGBS) and the aggregates, in a rotating mechanical concrete mixer for about 2 min. The alkaline activator solutions were concurrently added to the water and mixed for about 3 min until a uniformly mixed concrete was formed. The process was repeated for all the geopolymer mix designs with different A/P ratios. The consistency of the fresh concrete for each design mix, including the control mix, was determined in accordance with BS EN 12350-2: 2019 [49], using the slump test to determine their workability potential. Six concrete test specimens of a standard cube dimension (100 mm × 100 mm × 100 mm), two concrete cylinder of dimension 150 mm × 300 mm, and one concrete beam of dimension 100 mm × 100 mm × 500 mm were each prepared for the mix designs in accordance with BS EN 12390-2:2019 [50], for the determination of the unconfined compressive, tensile, and flexural strengths of each of the concrete design mixes [51]. The concrete test specimens were demoulded after 24 h and kept in a moist-curing tank at an ambient temperature of 20 ± 2 °C. Moist-curing was adopted over heat-curing for the geopolymer test specimens to simulate practical in situ concrete production and obtain concrete with a lower GWP (global warming potential) [30], although slag-based geopolymers perform better in compressive strength under elevated temperatures [52].

The hardened concrete density of the cubes and cylinder was determined for each mix after curing in accordance with BS EN 12390-7:2019 [53]. Three test specimens for the concrete cubes and two cylinders each for the design mixes were computed, and the average was recorded. At the end of the curing period, the compressive strength of the concrete cube specimen for each design mix was determined using a 2000 kN compression testing machine motorised with a Servo-Plus Progress control unit (YIM2000KNFMT/AH/44, MATEST, Begamo, Italy). Three test specimens for each design mixes were tested after 3, 7, 28, and 56 days for their compressive strength in accordance with BS EN 12390-3:2019 [54], and the averages were computed. Two concrete cylinders were tested in a 28-day tensile splitting strength test using a 2000 kN compression machine in compliance with BS EN 12390-6:2019 [55]. To determine the flexural strength, a four-point loading bending test was also carried out on a concrete beam specimen for each mix during the 28-day curing period. This was carried out using a 50-ton capacity Denison Universal Testing machine (89067/7223, Samuel Denison Limited, Leeds, UK) in accordance with BS EN 12390-5: 2019 [56], with the application of load at a constant rate increase of 10 kN/min on the specimen until failure.

## 4. Results and Discussion

### 4.1. Consistency of Fresh Concrete

**Figure 2:**
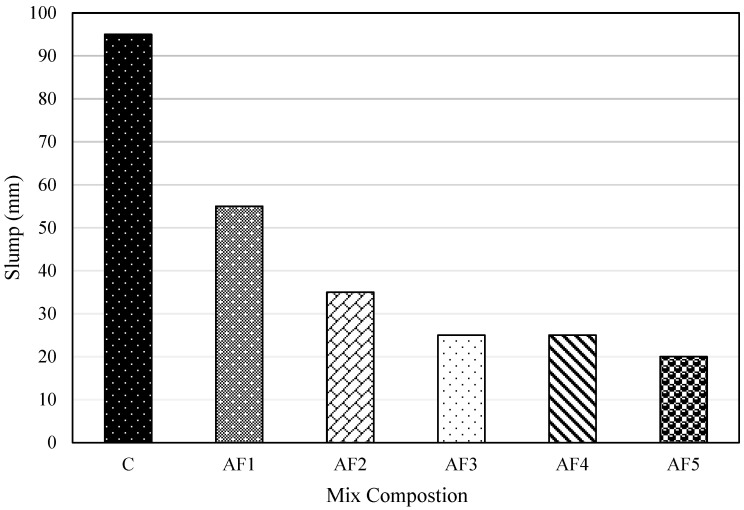
Consistency of concrete mixes measured—slump test.

Figure 2 shows the slump values of fresh concrete for all the mixes. The control concrete (C) attained a maximum slump value of 95 mm, and the geopolymer concrete mix AF5, with an A/P ratio of 0.5, recorded the lowest slump value of 20 mm, making OPC concrete more workable than the geopolymer concrete. Observation also showed that the OPC control mix obtained the maximum slump value among all the mix designs, thus being more workable than the geopolymer mix designs. Bellum et al. [57] obtained a similar trend in a study involving a GGBS fly ash-based geopolymer compared with OPC concrete, and an increase in the GGBS content influenced a lower workability outcome. This lower workability trend observed for the geopolymer formulation over PC concrete could be attributed to the rapid loss of workability from the quickened hydration reaction process usually provided by the activation of slag, against that of PC concrete, which has a lower initial rate of reaction [58]. GGBS, as a precursor in geopolymer formulation, has lower workability and flowability due to its irregular shape, which reduces its mobility during alkaline activation [59]. This is also because the activated GGBS geopolymer concrete entrains more air than the PC control concrete. Likewise, the highly viscous nature of the sodium silicate alkaline solution affects workability when used to activate GGBS to develop geopolymer concrete because it introduces a sticky characteristic into the fresh concrete and thus contributes to the lower workability of the geopolymer mix against the PC mix [30,60,61].

The AF1 geopolymer mix, with an A/P ratio of 0.1, recorded the maximum slump value of 55 mm among the geopolymer mixes, having a higher workability. The workability of the geopolymer mixes decreased from AF1 to AF5 with increasing A/P ratios, though AF3 and AF4 recorded the same result. AF1 is classified as having medium workability in the 50–89 mm range, while the rest of the geopolymer mixes that belonged to the class of alkaline-activated concrete, recorded low workability for values below 50 mm [61]. The workability of the geopolymer mixes decreased with increasing A/P ratios. This was opposed to the trend observed by Adeleke et al. [30], where slump values decreased with decreasing A/P ratios. For alkali-activated slag concrete, workability reduced with increasing alkali activator moduli [43]. Increasing the A/P ratio with an SSA:SH alkali solution ratio of 1:1 culminated in the increase in the volume of the activators while decreasing the GGBS. In addition, it is worth noting that the sticky and viscous effect of the alkaline activator whilst mixing the concrete was higher with a decreasing content of GGBS, causing a decrease in workability [30]. Also, as the A/P ratio increased with the dry solid (GGBS) decreasing, the rate of dissolution of the GGBS material and the formation of reactant gel products (hydration process) increased at an early stage, causing the initial slump to decrease and resulted in an increase in slump loss [62]. Thus, the more reactant products formed for increasing A/P ratios, the less the workability.

### 4.2. Density of Hardened Concrete

**Figure 3:**
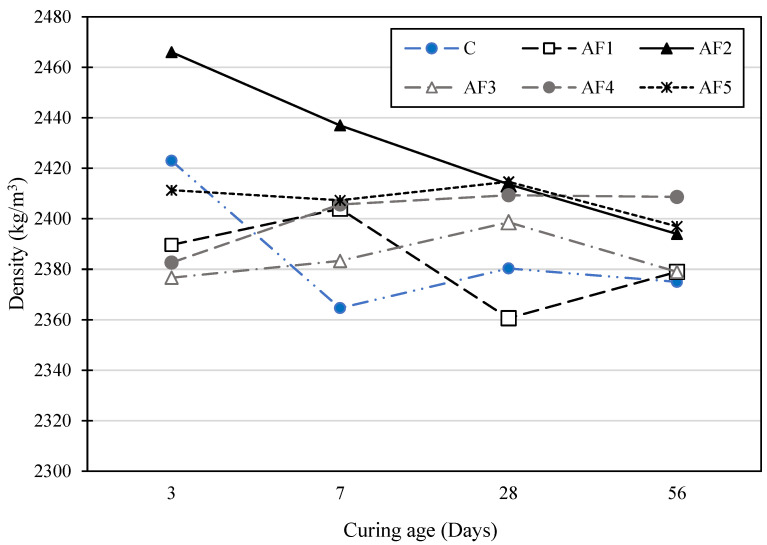
Hardened concrete density of test mix with time.

Figure 3 illustrates the average density results of the concrete mixes. The investigations show that mix AF2 recorded the highest density of 2466 kg/m^3^ on day 3 of moist-curing. However, the density measurement decreased progressively after 3 days, arriving to a value of 2394.0 kg/m^3^ after 56 days of moist-curing. The density of the 28-day-hardened concrete recorded for the control OPC concrete was 2380.3 kg/m^3^, against those of the geopolymer concrete specimens, which were in the range of 2360.7–2414.7 kg/m^3^. Apart from AF2, which registered a trend of decreasing densities over time, others showed variations in their density values. This trend was similarly observed by Nath and Sarker [63]. The rest of the specimens had mixed values with time, though with slight differences. The density values of the control mix design and the geopolymer mix designs were all within the required range for normal-weight concrete and complied with BS EN 206:2013+A2:2021 [64]. The changes in the density values with time for the different mixes could have been because of the different geopolymerisation evolution processes and water dissipation rates for each mix caused by the different A/P ratios [63]. However, Vijai et al. [65] suggested that variations in density values with time do not have much significance.

### 4.3. Concrete Strength Development

**Figure 4:**
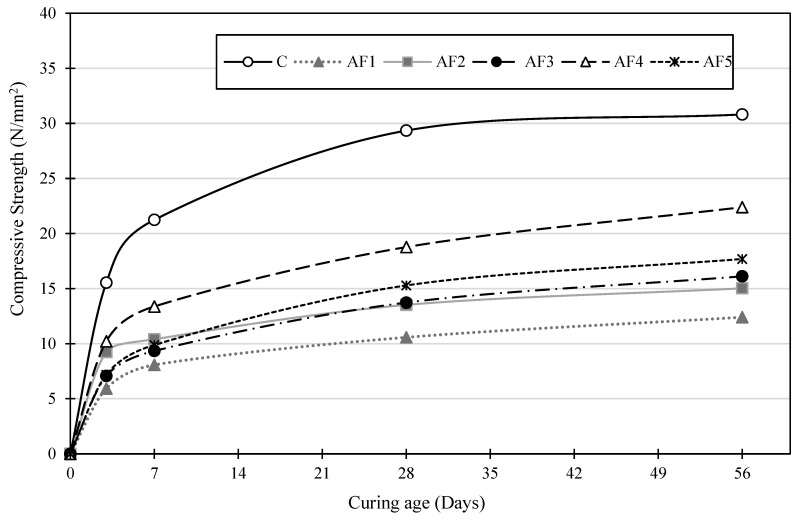
UCS development of the design mixes with time.

The average unconfined compressive strength (UCS) results for the concrete cube test of all the mix designs are illustrated in Figure 4. All the mix test specimens recorded an increasing UCS over the curing period, until 56 days. The control (OPC mix C) recorded the maximum UCS values of 15.5 MPa and 21 MPa after 3 and 7 days of curing, respectively, while mix AF4 achieved the highest values out of all the geopolymer mixes, recording UCS values of 10.2 MPa and 13.4 MPa after 3 and 7 days of curing. The AF1 test specimen, with an A/P ratio of 0.1, recorded the lowest UCS values of 5.9 MPa and 8.1 MPa after 3 and 7 days of curing, respectively. Alkali-activated slag is known to develop a rapid setting time and early strength [66,67]. Also, the SSA slows the initial reaction and leads to a low early strength for alkali-activated slag concrete compared to NaOH, which produces a high early strength that does not improve at later ages [68]. The low early strength could have partly been due to the SSA activator effect on the mix. The main reaction products for strength gain that resulted from the alkaline activation of the slag were crystalline calcium aluminium silicate-hydrated gel (C-A-S-H) and calcium silicate-hydrated gel (C-S-H (Calcium Silicate Hydrate)), together with sodium silicate-hydrated gel (N-A-S-H) [14,23,69]. The dissolution rate and leaching of the Al and Si in the GGBS material during the polymerisation process were influenced by the type and combination of alkaline solutions and their molar concentrations. Therefore, the combined alkaline solutions could have released fewer Ca ions from the precursor during the activation and dissolution processes to form the C-A-S-H gel responsible for the early strength build-up [70,71].

The control mix C recorded a higher 28-day UCS of 29.4 MPa than the 18.8 MPa value recorded for AF4, which had the maximum UCS out of the geopolymer mixes, and AF1 had the lowest 28-day UCS, 10.6 MPa. Sithole and Mashifana [72] recorded a compressive strength of 31.3 N/mm^2^ for a GGBS-based geopolymer brick with only a 10 M NaOH activator solution but an elevated curing temperature of 800 °C. Özdal et al. [73] also recorded a higher 28-day strength with a similar GGBS-based geopolymer design but cured at 80 °C for 24 h and then at ambient temperature for another 27 days. This shows that the hydration process could have been delayed by adopting the moist-curing method. The 56-day compressive strengths for all the mix designs increased, with the geopolymer mix designs obtaining a higher percentage increment from the 28-day compressive strength result than the control mix design with PC. Also, mix AF4 recorded the highest percentage increment of about 19%, recording a late strength of 22.4 MPa after 56 days, with mix C recording 30.8 MPa after 56 days from its 29.4 MPa value recorded after 28 days. The control mix achieved over 95% of its UCS within 56 days of curing, over a total of 28 days, with an expected designed UCS of 30 MPa for a mix ratio of 1:2:3.

The mechanical properties of geopolymer concrete are also influenced by the proportion of SiO_2_ in the binder, especially from Na_2_SiO_3_ [26]. The lower UCS of the geopolymer mixes could have been due to inadequate amounts of SiO_3_^2−^ provided by the pumice-derived Na_2_SiO_3_ solution to react with Ca ion-less C-A-S-H. The extra water added to the original geopolymer design to improve the workability [48] could have also impacted the UCS [29]. However, some studies indicate that water in slag-based geopolymer only influences the setting time and not the UCS [29]. Water in the pores only serves as a medium for the polymerisation reaction [27], but extra water changes the alkaline activator concentration and the alkalinity of the pore solution, extending the setting time and modifying the structure and the number of reactants to be formed [74]. Cui et al. [75] demonstrated that increasing water in a geopolymer mix increases the n(H_2_O: Na_2_Oeq) ratios, thus resulting in lower compressive strengths due to increased porosity and pore size. This is supported by Jeong et al. [76] for a GGBS geopolymer formulation that resulted in a higher early strength when the water content was reduced, leading to a subsequent reduction in porosity.

The geopolymer mix designs recorded an increasing UCS trend from AF1 to AF4, with increasing A/P ratios, and decreased from AF4 to AF5. A review by Shilar et al. [77] had an A/P ratio of 0.4 as the optimum design for the maximum UCS. Increasing the A/P ratio ensures the use of less aluminosilicate material (solids) and more alkaline solution (liquid). This allowed for a higher dissolution of the precursor species and the formation of more reactant products, which influenced the strength gain. When GGBS is activated with a blend of NaOH and Na_2_SiO_3_ alkaline solutions, the higher Na_2_O/SiO_2_ ratio as a percentage of the slag content causes an increase in the compressive strength [78]. The Na_2_O content increases with increasing A/P ratios, leading to a higher strength for the mix designs as the A/P ratio increases. The mix design AF5 recorded a 28-day strength lower than AF4 even though the A/P ratio was increased, the alkaline component was increased, and the precursor quantity was reduced; this could have been because of excess alkaline solution causing excess liquids and thus hindering the polymerisation process and reducing the strength [59]. The geopolymer concrete specimen experienced some efflorescent growth on the concrete cube surface. This efflorescent growth displayed on the geopolymer concrete cubes, also observed by Adeleke et al. [30], after the period of curing was due to the formation of bicarbonate (white) crystals from the reaction of excess alkali from the NaOH and CO_2_ but had no effect on the structural integrity of the concrete, apart from the aesthetics of the concrete [23]. This growth is associated with the excessive use of highly concentrated NaOH and KOH activator solutions, which could be reduced by a significant degree by adopting a geopolymer formulation with a Si/Al ratio of 1.5 [18].

### 4.4. Tensile Splitting Strength of Concrete Mix Designs

**Figure 5:**
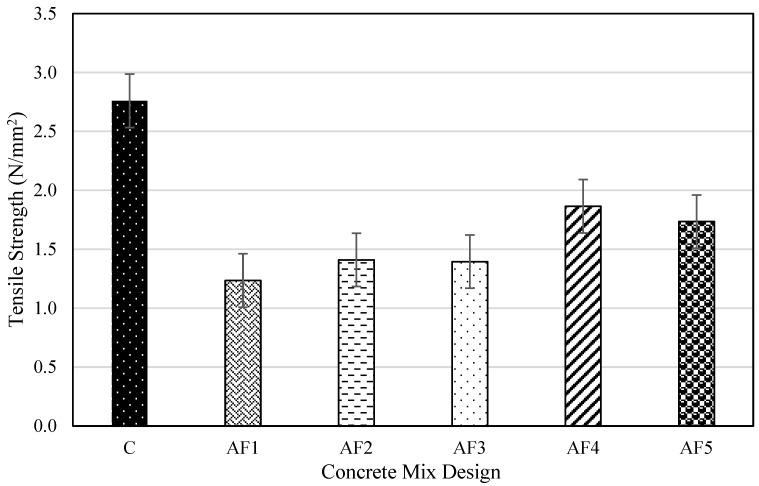
Splitting test results for the design mixes.

The tensile splitting strength results of the concrete cylinder specimen for all the mix designs after 28 days are displayed in Figure 5. The control mix C recorded the maximum tensile strength (TS) value of 2.76 N/mm^2^ among all the test mixes, making OPC higher in TS than the geopolymer concrete. As in other studies, the results obtained confirm that the TS values of the geopolymer formulation, as in the case of OPC concrete, are a fraction of the compressive strength value [79]. The lower tensile splitting strength of the geopolymer formulations than the PC concrete is opposed to the findings in other studies [30,57], which observed all geopolymer concretes to have a higher tensile splitting strength than PC concrete. Geopolymer formulations are expected to perform better in tension than PC concrete, though that tensile property depends on the composition of the geopolymer concrete [80]. The extra water added to the original design to improve workability could have also affected the geopolymerisation process, hence the lower TS for the geopolymer mixes.

The AF4 test specimen recorded the maximum tensile strength value, while AF1 registered the lowest tensile strength result of 1.24 N/mm^2^. The TS values for the geopolymer concrete increased from AF1 to AF4 with increasing A/P ratios, then decreased to AF5 with a slight decrease between AF2 and AF3. Thomas and Peethamparan [81] suggest that the sensitivity of the tensile splitting strength of AAM is affected by the curing conditions. The moist-curing condition employed for all the test specimens could have affected the expected tensile splitting strengths of the geopolymer concretes and even the PC concrete [82]. The predicted tensile splitting strength results for the geopolymer formulations and the OPC concrete mix using a model from Gaedicke et al. [83] yielded a higher value than that experimentally obtained. This is due to all the reasons ascribed to the case regarding the variation in compressive strength. This view is also shared by Adeleke et al. [30], who believe that the A/P ratio and sodium silicate-to-sodium hydroxide ratio equally impact the compressive and tensile splitting strengths.

### 4.5. Flexural Strength of Concrete Mix Designs

**Figure 6:**
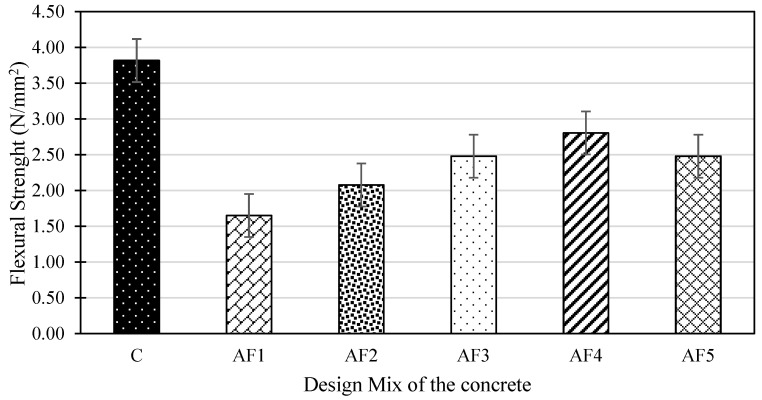
Maximum flexural strength for the design mixes.

The 28-day maximum flexural strength results of the beam specimen from the four-point flexural test are shown in Figure 6. The control OPC specimen recorded a flexural strength value of 3.82 N/mm^2^, higher than the value of the geopolymer concrete specimens. This was inconsistent with Nath and Sarker [63], who observed a higher value for geopolymer formulations than PC concrete beams, and could be due to the type of material and varied formulations used in the current research. Ansari et al. [84] also concluded, in a recent review, that the flexural strength of geopolymer concrete is comparable to that of OPC concrete, which could mean a lower or similar results, as evidenced in the current research. Regarding the geopolymer formulations, mix AF4 registered the maximum flexural strengths of 2.81 N/mm^2^, while AF1 recorded the lowest value of 1.65 N/mm^2^. In addition, mixes AF3 and AF5 both recorded similar flexural strength values of 2.48 N/mm^2^. It is worth noting that the flexural strength for geopolymer formulations increased with an increasing A/P ratio (AF1 to AF4) and then decreased for AF4. This trend is similar to the tensile strength performance for the geopolymer mixes. Therefore, flexural strength values correlate with tensile strength. Hence, the same reasons that influenced the lower compressive and tensile strengths could be applicable here.

## 5. Conclusions

The outcome of the present study suggests the practicability of using an SSA alkaline solution derived from pumice powder as one of the alkaline activators of a two-part geopolymer design for a GGBS-based geopolymer concrete for low-strength construction applications. The following conclusions can be drawn from this study.

1. The workability of the geopolymer formulations was lower than that of the OPC concrete. For the geopolymer formulations, workability was reduced with an increasing A/P ratio. This confirms the poor workability of GGBS-based geopolymers. This could be addressed by blending GGBS with another precursor like fly ash, which can improve the workability with less water added.
2. The hardened density of the concrete produced by the geopolymer was comparable and even slightly higher for most geopolymer formulations (AF2, AF3, AF4, AF5) on day 28 than the control PC concrete. This could be because of the dense and lower-porosity concrete formed by the activating solutions.
3. The alkaline precursor ratio influenced the geopolymerisation and mechanical properties of the concrete. The GGBS-based geopolymer concrete improved in terms of mechanical properties, including compressive strength, tensile splitting strength, and flexural strength, with an increasing A/P ratio up to an A/P ratio of 0.4. It then reduced in performance after increasing the A/P ratio. It thus shows that the optimum design for the geopolymer formulation using the SSA derived from PP was at an A/P ratio of 0.4.
4. The mechanical performance of the geopolymer concrete designs was lower than that of the control OPC concrete. The control concrete achieved a 28-day compressive strength of 29.4 MPa while the optimum geopolymer design recorded values of 18.8 MPa after 28 days and 22.4 MPa (19% increase) after 56 days, against OPC concrete that recorded 30.8 MPa on day 56 (5% increase). This shows that the dissolution effect of the extra water in the geopolymer mix might have affected the rate of geopolymerisation. The tensile splitting strength and the flexural strength results recorded for the geopolymer formulations, in contrast with the control concrete, observed a similar trend to compressive strength of all the mix designs studied.
5. This study shows that the SSA-derived alkaline solution from pumice powder has the potential for developing geopolymer concrete even with lower results. This is because geopolymerisation is a complex process, and the resulting mechanical performance is influenced by all the parameters used in the concrete’s formulation. The AF4 mix could still be used in low-strength construction applications.
6. Further research can be carried out to assess the durability of the developed concrete, coupled with a detailed carbon capture analysis/life cycle assessment.

## Figures and Tables

**Table 1:** Oxide compositions of OPC, GGBS, and pumice [30,45].

Oxides	Compositions (%)		
	OPC	GGBS	Pumice
CaO	61.49	37.99	0.8
MgO	3.54	8.78	0.1
SiO_2_	18.84	35.54	76.2
Al_2_O_3_	4.77	11.46	13.5
Na_2_O	0.02	0.37	1.6
P_2_O_5_	0.1	0.02	1.8
Fe_2_O_3_	2.87	0.42	1.1
Mn_2_O_3_	0.05	0.43	-
K_2_O	0.57	0.43	-
TiO_2_	0.26	0.7	0.2
V_2_O_5_	0.06	0.04	-
BaO	0.05	0.09	-
SO_3_	3.12	1.54	-
Loss on ignition	4.3	2	-

**Table 2:** Some physical and mechanical properties of pumice [45].

Other Properties	Pumice
Chemical Name	Amorphous Aluminium Silicate
Hardness (MOHS)	6
pH	7.2
Radioactivity	None
Loss on Ignition (LOI)	0.05
Softening Point	900 °C
Water-Soluble Substances	0.0015
Acid-Soluble Substances	0.029
Reactivity	Inert
Appearance	White powder (GE brightness of 84)

MOHS—measure of hardness (mechanical property).

**Table 3:** Geometrical, mechanical, and physical properties of the aggregates [30].

Properties	Fine Aggregate (Sand)	Coarse Aggregate (10 mm)	Coarse Aggregate (20 mm)
Shape index (%)	-	12	32
Impact value	-	18	12
Saturated density (kg/m^3^)	2820	2680	1330
Dry density (kg/m^3^)	2710	2570	1420
Water absorption (%)	0.85	1.5	12.8
Flakiness index (%)	-	23	37

**Table 4:** Mix design and material quantities used to produce concrete test specimens.

Mix Code	Elaborated Abbreviation	Concrete Binder						W (L)	Aggregates (kg)		
		OPC (kg)	Geopolymer Binder						FA	Coarse Aggregate	
			GGBS (kg)	A/P	SS:SH	Activator (mL)					
				Ratio		SS	SH			10 mm	20 mm
C	OPC (Control 1)	8.9	-	-	-	-	-	3.7	7.9	8.8	17.8
AF1	AF1-AP0.1-1SSA:SH	-	8.1	0.1	01:01	275	275	3.2	7.9	8.8	17.8
AF2	AF2-AP0.2-1SSA:SH	-	7.4	0.2	01:01	505	505	2.7	7.9	8.8	17.8
AF3	AF3-AP0.3-1SSA:SH	-	6.8	0.3	01:01	699	699	2.3	7.9	8.8	17.8
AF4	AF4-AP0.4-1SSA:SH	-	6.3	0.4	01:01	865	865	2.0	7.9	8.8	17.8
AF5	AF5-AP0.5-1SSA:SH	-	5.9	0.5	01:01	1010	1010	1.7	7.9	8.8	17.8

OPC—Ordinary Portland Cement; GGBS—ground granulated blast-furnace slag; A/P—activator/precursor ratio; SS:SH—sodium silicate-to-sodium hydroxide ratio; FA—fine aggregate; and W—water.

## Data Availability

Data are contained within the article.

## References

[1] Provis J.L., Van Deventer J.S.J. (2009). Geopolymers: Structures, Processing, Properties and Industrial Applications.

[2] Gunning J.G. (1983). Concrete Technology (Level 4).

[3] Oti J.E., Kinuthia J.M., Adeleke B.O., Billong N. (2020). Durability of Concrete Containing PFA-GGBS By-Products. J. Civ. Eng. Constr..

[4] Cement IEA. https://www.iea.org/reports/cement.

[5] UratanI J.M., Griffiths S. (2023). A Forward Looking Perspective on the Cement and Concrete Industry: Implications of Growth and Development in the Global South. Energy Res. Soc. Sci..

[6] Awoyera P., Adesina A. (2019). A Critical Review on Application of Alkali Activated Slag as a Sustainable Composite Binder. Case Stud. Constr. Mater..

[7] Heath A., Paine K., Goodhew S., Ramage M. (2013). The Potential for Using Geopolymer Concrete in the UK. Proc. Inst. Civ. Eng. Constr. Mater..

[8] Oti J. (2018). Engineering Properties of Concrete made with Pulverised Fly Ash. Int. J. Mech. Prod. Eng..

[9] Zhou X., Slater J.R., Wavell S.E., Oladiran O. (2012). Effects of PFA and GGBS on Early-Ages Engineering Properties of Portland Cement Systems. J. Adv. Concr. Technol..

[10] Dhir R.K., McCarthy M.J., Magee B.J. (1998). Impact of BS EN 450 PFA on Concrete Construction in the UK. Constr. Build. Mater..

[11] Swamy R.N. (1986). Cement Replacement Materials.

[12] Singh B., Ishwarya G., Gupta M., Bhattacharyya S.k. (2015). Geopolymer Concrete: A review of some recent developments. Constr. Build. Mater..

[13] Krivenko P.V., Kovalachuk G. (2007). Directed Synthesis of Alkaline Aluminosilicate Minerals in a Geocement Matrix. Mater. Sci..

[14] Pacheco-Torgal F., Labrincha J., Leonelli C., Palomo A., Chindaprasit P. (2015). Handbook of Akali-Activated Cements, Mortars and Concretes.

[15] Aldred J., Day J. Is Geopolymer Concrete a Suitable Alternative to Traditional Concrete. Proceedings of the 37th Conference on Our World in Concrete and Structures.

[16] Chanh N.V., Trung B.D., Tuan D.V. Recent Research Geopolymer Concrete. Proceedings of the 3rd ACF International Conference-ACF/VCA.

[17] Duxson P., Fernández-Jiménez A., Provis J.L., Lukey G.C., Palomo A., Van Deventer J.S.J. (2007). Geopolymer technology: The current state of the art. J. Mater. Sci..

[18] Singh N.B., Kumar M., Rai S. (2020). Geopolymer cement and concrete: Properties. Mater. Today Proc..

[19] Shobeiri V., Bennett B., Xie T., Visintin P. (2021). A Comprehensive Assessment of the Global Warming Potential of Geopolymer Concrete. J. Clean. Prod..

[20] Turner L.K., Collins F.G. (2013). Carbon Dioxide Equivalent (CO_2_-e) Emissions: A Comparison Between Geopolymer and OPC Cement Concrete. Constr. Build. Mater..

[21] Duxson P., Provis J.L., Lukey G.C., Van Deventer J.S.J. (2007). The Role of Inorganic Polymer Technology in the Development of ‘Green Concrete’. Cem. Concr. Res..

[22] Davidovits J. (1994). Properties of Geopolymer Concrete. First International Conference on Alkaline Cements and Concrete. Sci. Inst. Bind. Mater..

[23] Provis J.L., Van Deventer J.S. (2014). Alkali Activated Materials State-of-the-Art Report, RILEM TC 224-AAM.

[24] Zhang H.-Y., Liu J.-C., Wu B. (2021). Mechanical Properties and Reaction Mechanism of One-Part Geopolymer Mortars. Constr. Build. Mater..

[25] Alsalman A., Assi L.N., Kareem R.S., Carter K., Ziehl P. (2021). Energy and CO_2_ Emission Assessments of Alkali-Activated Concrete and Ordinary Portland Cement Concrete: A Comparative Analysis of Different Grades of Concrete. Clean. Environ. Syst..

[26] Bajpai R., Choudhary K., Srivastava A., Sangwan K.S., Singh M. (2020). Environmental Impact Assessment of Fly Ash and Silica Fume Based Geopolymer Concrete. J. Clean. Prod..

[27] Amran M., Al-Fakih A., Chu S.H., Fediuk R., Haruna S., Azevedo A., Vatin N. (2021). Long-Term Durability Properties of Geopolymer Concrete: An In-depth Review. Case Stud. Constr. Mater..

[28] Jamieson E., McLellan B., Riessen A.v., Nikraz H. (2015). Comparison of Embodied Energies of Ordinary Portland Cement with Bayer-Derived Geopolymer Products. J. Clean. Prod..

[29] Billong N., Oti J., Kinuthia J. (2021). Using Silica Fume Based Activator in Sustainable Geopolymer Binder for Building Application. Constr. Build. Mater..

[30] Adeleke B.O., Kinuthia J.M., Oti J., Ebailila M. (2023). Physico-Mechanical Evaluation of Geopolymer Concrete Activated by Sodium Hydroxide and Silica Fume-Synthesised Sodium Hydroxide Solution. Materials.

[31] Kamseu E., Moungam L.M.B., Cannio M., Billong N., Chaysuwan D., Melo U.C., Leonelli C. (2017). Substitution of Sodium Silicate with Rice Husk Ash-NaOH Solution in Metakaolin Based Geopolymer Cement Concerning Reduction in Global Warming. J. Clean. Prod..

[32] Rajan H.S., Kathirvel P. (2021). Sustainable Development of Geopolymer Binder using Sodium Silicate Synthesized from Agricultural Waste. J. Clean. Prod..

[33] Figueiredo R.A.M., Brandão P.R.G., Soutsos M., Henriques A.B., Fourie A., Mazzinghy D.B. (2021). Producing Sodium Silicate Powder from Iron Ore Tailings for use as an Activator in One-Part Geopolymer Binders. Mater. Lett..

[34] Tong K.T., Vinai R., Soutsos M.N. (2018). Use of Vietnamese Rice Husk Ash for the Production of Sodium Silicate as the Activator for Alkali-Activated Binders. J. Clean. Prod..

[35] Fitzsimmons C. How Is Pumice Formed?. https://sciencing.com/pumice-formed-5232410.html.

[36] Rashad A.M. (2021). An Overview of Pumice Stone as Cementitious Material-The Best Manual for Civil Engineer. Silicon.

[37] Kabay N., Mert M., Miyan N., Omur T. (2021). Pumice as Precursor in Geopolymer Paste and Mortar. J. Civ. Eng. Constr..

[38] Safari Z., Kurda R., Al-Hadad B., Mahmood F., Tapan M. (2020). Mechanical Characteristics of Pumice-Based Geopolymer Paste. Resour. Conserv. Recycl..

[39] Nematollahi B., Sanjayan J., Shaikh F.U.A. (2015). Synthesis of Heat and Ambient Cured One-Part Geopolymer Mixes with Different Grades of Sodium Silicate. Ceram. Int..

[40] Gokçe H.S., Tuyan M., Nehdi M.L. (2021). Alkali-Activated and Geopolymer Materials Developed Using Innovative Manufacturing Techniques: A Critical Review. Constr. Build. Mater..

[41] Provis J.L., Van Deventer J.S.J. (2013). Alkali Activated Materials: State-of-The-Art Report (RILEM TC 224-AAM).

[42] (2011). Cement. Part 1: Composition, Specifications and Conformity Criteria for Common Cements.

[43] Law D.W., Adam A.A., Molyneaux T.K., Patnaikuni I. (2012). Durability Assessment of Alkali Activated Slag (AAS) Concrete. Mater. Struct..

[44] (2006). Ground Granulated Blast Furnace Slag for Use in Concrete, Mortar and Grout—Part 1: Definitions, Specifications and Conformity Criteria.

[45] Mat M. (2023). GEOLOGYSCIENCE: Pumice. ed. https://geologyscience.com/rocks/igneous-rocks/extrusive-igneous-rocks/pumice/?amp.

[46] (2008). Aggregates for Concrete.

[47] (2016). Concrete—Specification, Performance, Production and Conformity.

[48] Bernal S.A., Provis J.L., van Deventer J.S.J., Basheer P.A.M. (2018). Impact of water content on the performance of alkali-activated slag concretes. Durability of Concrete Structures, Proceedings of the ICDCS2018: 6th International Conference on Durability of Concrete Structures, Leeds, UK, 18–20 July 2018.

[49] (2019). Testing Fresh Concrete. Part 1: Slump Test.

[50] (2019). Testing Hardened Concrete. Part 2: Making and Curing Specimens for Strength Tests.

[51] (2019). Testing Hardened Concrete. Making and Curing Specimens for Strength Tests.

[52] Yunsheng Z., Wei S., Qianli C., Lin C. (2007). Synthesis and Heavy Metal Immobilization Behaviors of Slag Based Geopolymer. J. Hazard. Mater..

[53] (2019). Testing Hardened Concrete. Density of Hardened Concrete.

[54] (2019). Testing Hardened Concrete. Part 3: Compressive Strength of Test Specimens.

[55] (2009). Testing Hardened Concrete. Tensile Splitting Strength of Test Specimens.

[56] (2019). Testing Hardened Concrete. Flexural Strength of Test Specimens.

[57] Bellum R.R., Muniraj K., Madduru S.R.C. (2020). Exploration of Mechanical and Durability Characteristics of Fly Ash GGBFS Based Green Geopolymer Concrete. Appl. Sci..

[58] Collins F.G., Sanjayan J.G. (1999). Workability and Mechanical Properties of Alkali Activated Slag Concrete. Cem. Concr. Res..

[59] Nath P., Sarker P.K. (2014). Effect of GGBFS on Setting, Workability and Early Strength Properties of Fly Ash Geopolymer Concrete Cured in Ambient Condition. Constr. Build. Mater..

[60] Yang T., Zhu H., Zhang Z., Gao X., Zhang C., Wu Q. (2018). Effect of Fly Ash Microsphere on the Rheology and Microstructure of Alkali Activated Fly Ash/Slag Pastes. Cem. Concr. Res..

[61] Fang G., Ho W.K., Tu W., Zhang M. (2018). Workability and Mechanical Properties of Alkali-Activated Fly Ash-Slag Concrete Cured at Ambient Temperature. Constr. Build. Mater..

[62] Jia R., Wang Q., Luo T. (2022). Understanding the Workability of Alkali-Activated Phosphorus Slag Pastes: Effects of Alkali Dose and Silicate Modulus on Early-Age Hydration Reactions. Cem. Concr. Compos..

[63] Nath P., Sarker P.K. (2017). Flexural Strength and Elastic Modulus of Ambient-Cured Blended Low-Calcium Fly Ash Geopolymer Concrete. Constr. Build. Mater..

[64] (2021). Concrete Specification, Performance, Production and Conformity.

[65] Vijai K., Kumutha R., Vishnuram B.G. (2010). Effect of Types of Curing on Strength of Geopolymer Concrete. Int. J. Phys. Sci..

[66] Li X., Wang Z., Jiao Z. (2013). Influence of Curing on the Strength Development of Calcium-Containing Geopolymer Mortar. Materials.

[67] Aziz I.H., Abdullah M.M.A.B., Salleh M.A.A.M., Azimi E.A., Chaiprapa J., Sandu A.V. (2020). Strength Development of Solely Ground Granulated Blast Furnace Glag Geopolymers. Constr. Build. Mater..

[68] Haha M.B., Lothenbach B., Saout G.L., Winnefeld F. (2012). Influence of Slag Chemistry on the Hydration of Alkali-Activated Blast-Furnace Slag—Part II: Effect of Al_2_O_3_. Cem. Concr. Res..

[69] Thunuguntla C.S., Gunneswara Rao T.D. (2018). Effect of Mix Design Parameters on Mechanical and Durability Properties of Alkali Activated Slag Concrete. Constr. Build. Mater..

[70] Zhu X., Zhang M., Yang K., Yu L., Yang C. (2020). Setting Behaviours and Early-Age Microstructures of Alkali-Activated Ground Granulated Blast Furnace Slag (GGBS) from Different Regions in China. Cem. Concr. Compos..

[71] Farhan K.Z., Johari M.A.M., Demirbog R. (2020). Assessment of Important Parameters Involved in the Synthesis of Geopolymer Composites: A Review. Constr. Build. Mater..

[72] Sithole N.T., Mashifana T. (2020). Geosynthesis of Building and Construction Materials through Alkaline Activation of Granulated Blast Furnace Slag. Constr. Build. Mater..

[73] Özdal M., Karakoç M.B., Özcan A. (2021). Investigation of the Properties of Two Different Slag-Based Geopolymer Concretes Exposed to Freeze–Thaw Cycles. Struct. Concr. J..

[74] Provis J.L., van Deventer J.S.J. (2007). Geopolymerisation Kinetics. 2. Reaction Kinetic Modelling. Chem. Eng. Sci..

[75] Cui Y.-l., Wang D., Wang Y., Sun R., Rui Y. (2019). Effects of the n(H_2_O: Na_2_Oeq) Ratio on the Geopolymerization Process and Microstructures of Fly Ash-Based Geopolymers. J. Non-Cryst. Solids.

[76] Jeong Y., Oh J.E., Jun Y., Park J., Ha J.-h., Sohn S.G. (2016). Influence of Four Additional Activators on Hydrated-Lime [Ca(OH)_2_] Activated Ground Granulated Blast-Furnace Slag. Cem. Concr. Compos..

[77] Shilar F.A., Ganachari S.V., Patil V.B., Khan T., Dawood Abdul Khadar M.Y.S. (2022). Molarity activity effect on mechanical and microstructure properties of geopolymer concrete: A review. Case Stud. Constr. Mater..

[78] Mohamed O., Khattab R., Alzo’ub A.K. (2019). Factors Affecting Compressive Strength Development in Alkali-activated Slag Concrete. IOP Conf. Ser. Mater. Sci. Eng..

[79] Rangan B.V. (2014). Geopolymer concrete for environmental protection. Indian Concr. J..

[80] Asghar R., Khan M.A., Alyousef R., Javed M.F., Ali M. (2023). Promoting the Green Construction: Scientometric Review on the Mechanical and Structural Performance of Geopolymer Concrete. Constr. Build. Mater..

[81] Thomas R.J., Peethamparan S. (2015). Alkali-Activated Concrete: Engineering Properties and Stress–Strain Behavior. Constr. Build. Mater..

[82] Vora P.R., Dave U.V. (2013). Parametric Studies on Compressive Strength of Geopolymer Concrete. Procedia Eng..

[83] Gaedicke C., Torres A., Huynh K.C., Marines A. (2016). A Method to Correlate Splitting Tensile Strength and Compressive Strength of Pervious Concrete Cylinders and Cores. Constr. Build. Mater..

[84] Ansari M.A., Shariq M., Mahdi F. (2023). Structural behavior of reinforced geopolymer concrete beams—A review. Mater. Today Proc..

